# Expanding the parameter space of anodal transcranial direct current stimulation of the primary motor cortex

**DOI:** 10.1038/s41598-019-54621-0

**Published:** 2019-12-03

**Authors:** Desmond Agboada, Mohsen Mosayebi Samani, Asif Jamil, Min-Fang Kuo, Michael A. Nitsche

**Affiliations:** 1 Department of Psychology and Neurosciences, Leibniz Research Centre for Working Environment and Human Factors, Dortmund, Germany; 2 International Graduate School of Neuroscience (IGSN), Ruhr University Bochum, Bochum, Germany; 3 Institute of Biomedical Engineering and Informatics, Ilmenau University of Technology, Ilmenau, Germany; 4 Department of Neurology, University Hospital Bergmannsheil, Bochum, Germany

**Keywords:** Motor cortex, Neurophysiology

## Abstract

Size and duration of the neuroplastic effects of tDCS depend on stimulation parameters, including stimulation duration and intensity of current. The impact of stimulation parameters on physiological effects is partially non-linear. To improve the utility of this intervention, it is critical to gather information about the impact of stimulation duration and intensity on neuroplasticity, while expanding the parameter space to improve efficacy. Anodal tDCS of 1–3 mA current intensity was applied for 15–30 minutes to study motor cortex plasticity. Sixteen healthy right-handed non-smoking volunteers participated in 10 sessions (intensity-duration pairs) of stimulation in a randomized cross-over design. Transcranial magnetic stimulation (TMS)-induced motor-evoked potentials (MEP) were recorded as outcome measures of tDCS effects until next evening after tDCS. All active stimulation conditions enhanced motor cortex excitability within the first 2 hours after stimulation. We observed no significant differences between the three stimulation intensities and durations on cortical excitability. A trend for larger cortical excitability enhancements was however observed for higher current intensities (1 vs 3 mA). These results add information about intensified tDCS protocols and suggest that the impact of anodal tDCS on neuroplasticity is relatively robust with respect to gradual alterations of stimulation intensity, and duration.

## Introduction

Neuroplasticity is the structural and functional modification of synaptic connections in response to internal or external stimuli. It is involved in cognitive and behavioural functions such as learning and memory formation in healthy organisms, but also in restitution of functions after brain lesions. In neurological and psychiatric diseases moreover, pathological alterations of neuroplasticity play a role. Thus, the exploration of mechanisms, and consequences of neuroplasticity is relevant. Modern intervention techniques have endowed us with the ability to induce neuroplasticity in the intact human brain non-invasively. These non-invasive brain stimulation (NIBS) tools, which include transcranial magnetic stimulation (TMS), and transcranial electric stimulation (tES) have enhanced our understanding of functional relationships between brain physiology and behaviour^1^. Transcranial direct current stimulation (tDCS) is one tES technique which has been shown to modulate cognitive functions in healthy humans^2^, and to alter psychiatric and neurological symptoms in patients via induction of plasticity^3–7^.

tDCS induces polarity-dependent neuroplastic changes in the brain, which can last for hours depending on the dosage of stimulation. In the primary motor cortex, an enhancement of cortical excitability is observed when the anode is placed over the target region with stimulation intensities for up to 2 mA, and stimulation durations for up to 20 min, and an excitability reduction with the cathode over the target region for a stimulation intensity of 1 mA and an electrode size of 35 cm^2^ ^8–10^. Unlike other NIBS techniques, such as repetitive TMS, tDCS uses weak direct currents to induce gradual changes of the resting membrane potential of cortical neurons in a polarity dependent manner – anodal stimulation leads to a subthreshold depolarization whereas cathodal stimulation leads to hyperpolarization of neuronal compartments critical for the respective excitability, and neuronal activity alterations^11,12^.

In conventional tDCS, two rubber electrodes covered in a saline-soaked sponge are placed on the scalp – one over the target area, and the other over a remote region^8^. Direct current passes through the skull and cerebrospinal fluid before reaching the brain. Whereas the primary effects of tDCS are due to neuronal membrane polarization, after-effects are caused by a modification of synaptic strength related to long-term potentiation (LTP) and long-term depression (LTD). tDCS-induced plasticity depends on glutamatergic mechanisms. It is calcium-, N-methyl-D-aspartate (NMDA) receptor-^13–15^, and α-amino-3-hydroxy-5-methyl-4-isoxazoleprionic acid (AMPA) receptor-dependent^16,17^. A concomitant reduction of GABA might serve as a gating mechanism^18,19^. For calcium-dependent plasticity, the amount of calcium influx determines the type of plasticity observed^20^. Whereas relatively low calcium concentration results in LTD, a high amount of calcium results in LTP. Between these concentrations, a no man’s land, or transition zone does exist, which results in no plasticity^21^. Excessive calcium concentration results likewise in no plasticity due to counteracting mechanisms, which include activation of potassium channels^22^.

Within the parameter space of 0.2–1 mA current intensity, and 5–13 minutes duration, previous studies have shown stronger and longer after-effects induced by stronger and longer stimulation^8,9^. However, some non-linear relationships between stimulation parameters and excitability alterations have been reported for stronger and longer-lasting stimulation protocols^23,24^, which are compatible with the above-mentioned calcium hypothesis. These recent studies stress the relevance of a systematic titration of tDCS parameters, to identify protocols that are resulting in stable and unidirectional effects.

The parameter space of tDCS however is large, with different parameters contributing to the direction and magnitude of after-effects. Intensity and duration of stimulation are two critical parameters. This notwithstanding, stimulation protocols have not systematically been further developed during the last years, with stimulation intensity and duration between 0.5–2 mA, and 10–30 minutes in most studies. Some study results suggest that increasing the current intensity does not necessarily lead to longer neuroplastic changes or treatment outcomes, or even convert the direction of after-effects^23–25^. Recently, Jamil and colleagues systematically evaluated the effect of four tDCS intensities - 0.5, 1, 1.5, and 2 mA – applied for the duration of 15 minutes, on motor cortical excitability, and found no significant differences in after-effects induced by these intensities for anodal tDCS^26^. Apart from the intensity of tDCS, stimulation duration also affects the amount of induced neuroplasticity^9^. When the duration of 1 mA anodal tDCS was doubled from 13 minutes to 26 minutes, stimulation did induce no excitability enhancement, but diminution, showing a dependence of directionality of tDCS-induced after-effects on the duration of stimulation^24^. This might be an effect of the above-mentioned calcium dynamics, where longer stimulation could lead to an overflow of calcium at the synapse, setting off a reversal of potentiation^21^. It remains to be established how different combinations of intensity and duration of stimulation affect cortical excitability, especially when current intensities and durations are increased beyond those commonly used. This is especially important because of non-linear dose-effect relations, as described above.

This study aims to expand and investigate systematically the parameter space of anodal tDCS with respect to current intensity and stimulation duration, to extend systematic information on optimally suited stimulation protocols for neuroplasticity induction based on the primary motor cortex model in healthy adults. Anodal tDCS was administered with three current intensities – 1, 2, and 3 mA, for three different durations – 15, 20, and 30 minutes. Cortical excitability changes were monitored with TMS-induced motor evoked potentials (MEP) as an index of neuroplasticity. It was hypothesized that anodal tDCS parameters might non-linearly correlate with stimulation after-effects. Previous studies have shown that anodal tDCS intensities up to 2 mA and durations up to 20 min result in excitability enhancements^23,27^, while longer stimulation led to a conversion of the polarity of after-effects^24^. We furthermore hypothesized that this reversal of excitability from enhancement to diminution with longer stimulation duration might also be observed for stronger intensities of stimulation, due to the calcium-dependency of induced plasticity.

## Results

**Figure 1:**
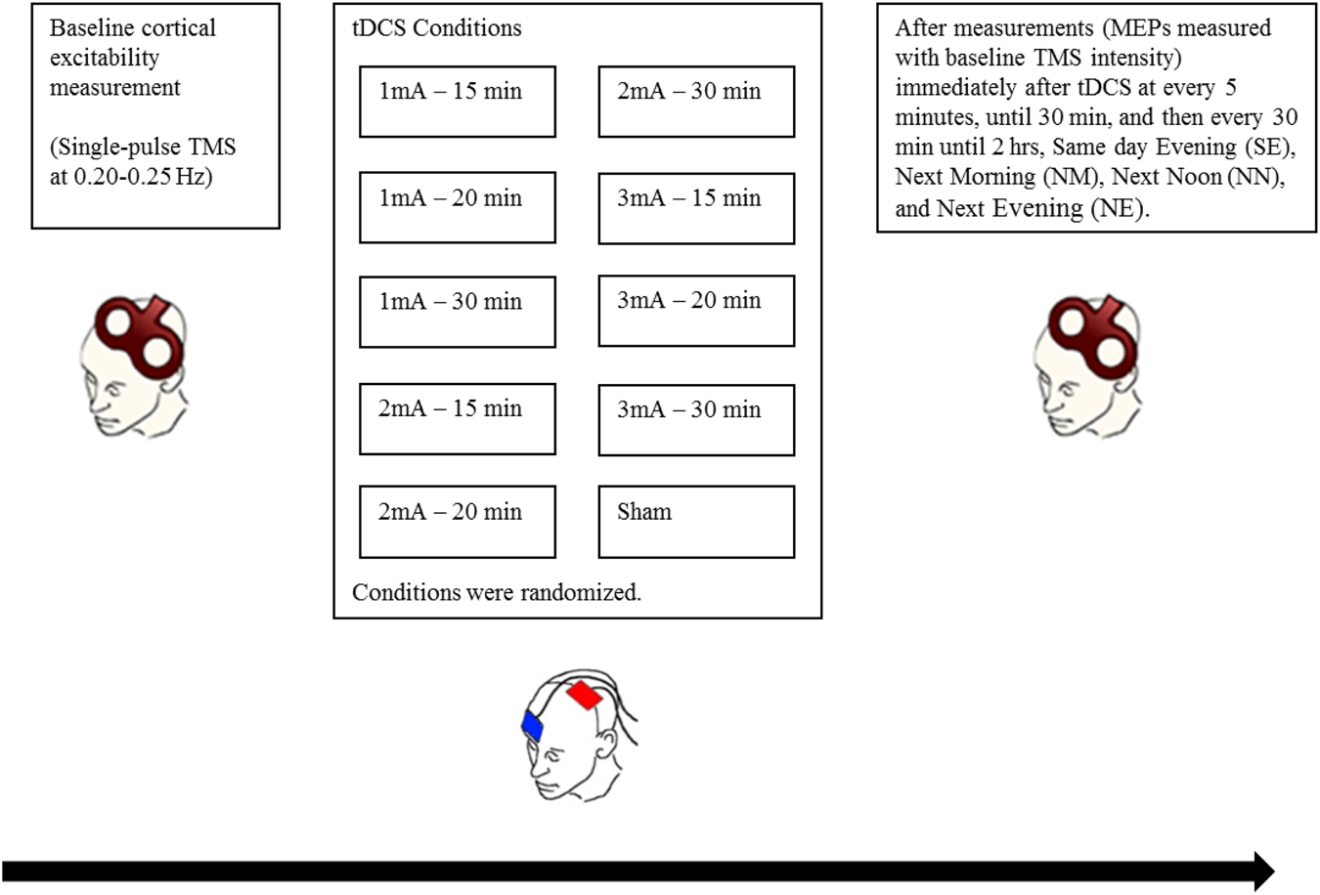
Diagrammatic representation of the experimental procedure. Each participant took part in 10 sessions of the experiment, with a minimum one-week inter-session interval to prevent carry-over effects. A condition as defined here refers to Intensity-Duration pairs (eg. 1 mA–30 min). All conditions were randomized. Each experimental session started with the identification of the ‘hotspot’ of the ADM, and the TMS intensity which resulted in a MEP amplitude of approximately 1 mV (baseline TMS intensity) was determined. MEPs were then recorded as a baseline excitability measure, after which tDCS was applied. Immediately after tDCS, MEPs were again recorded with the same baseline TMS stimulus intensity every 5 min until 30 min, every 30 min until 2 hr, SE, NM, NN, and NE (modified from Jamil^26^, with permission of The Journal of Physiology*, John Wiley and Sons*).

Data analysis was based on data collected from 16 participants. Each subject took part in 10 sessions of the experiment, with each session separated by at least one week (Fig. 1). For each session, 15 measurements of cortical excitability were made – one measurement before tDCS (baseline excitability), and 14 measurements after tDCS (after-effects) (Fig. 1).

Apart from slight tingling, burning sensations, and redness of the skin which are commonly reported in tDCS experiments^28^, all subjects tolerated the stimulation well. Side effects did not last more than 30 minutes after stimulation. Side effects did not differ between active and sham stimulation conditions. Blinding was not compromised; in no condition participants guessed the correct intensity more frequently than expected by chance level. The results of the analyses of side-effects and blinding data are presented in Tables 1–7 of the Supplementary Material.

Baseline MEPs as well as baseline TMS intensity were not significantly different between sessions (Table 1).

**Table 1:** Baseline MEPs and TMS Intensity for all 10 conditions.

Condition	Baseline MEP (mV)	Baseline TMS intensity (% MSO)
Sham	1.03 ± 0.11	61.62 ± 13.72
1 mA–15 min	1.03 ± 0.09	62.09 ± 12.87
1 mA–20 min	1.00 ± 0.09	62.50 ± 14.30
1 mA–30 min	0.98 ± 0.08	60.94 ± 14.71
2 mA–15 min	1.02 ± 0.09	61.65 ± 13.31
2 mA–20 min	0.98 ± 0.07	61.78 ± 14.03
2 mA–30 min	0.98 ± 0.08	61.84 ± 14.23
3 mA–15 min	1.05 ± 0.11	61.56 ± 13.34
3 mA–20 min	1.01 ± 0.09	62.31 ± 13.22
3 mA–30 min	1.02 ± 0.09	61.03 ± 13.44

MEP and TMS Intensity values are reported as mean ± standard deviation. (MSO: maximum stimulator output of TMS). The one-way ANOVA results showed no significant main effect of session for baseline MEP (F_(9, 135)_ = 1.146, η^2^p = 0.071, p = 0.335), and TMS intensity (F_(3.469, 52.028)_ = 0.634, η^2^p = 0.041, p = 0.619).

### No significant impact of stimulation intensity and duration on the effects of tDCS

We investigated the influence of tDCS parameters – intensity, and duration - on cortical excitability. These analyses were conducted without the sham stimulation condition, which only included one intensity/duration combination.

The three-way repeated measures ANOVA conducted over all 15 time bins shows no significant main effect of Intensity (F_(2,28)_ = 1.710, df = 2, η^2^_p_ = 0.109, p = 0.199) and Duration (F_(2,28)_ = 2.278, η^2^_p_ = 0.140, p = 0.121), but a significant main effect of Time (F_(3.962,55.475)_ = 4.614, η^2^_p_ = 0.248, p = 0.003). A trend-wise effect was identified for the Intensity and Time interaction (F_(28,392)_ = 1.471, η^2^_p_ = 0.095, p = 0.060). There were however no significant interactions between Intensity and Duration (F_(4,56)_ = 0.140, η^2^_p_ = 0.010, p = 0.967), Duration and Time (F_(28,392)_ = 0.791, η^2^_p_ = 0.053, p = 0.770), and Intensity, Duration, and Time (F_(56, 784)_ = 1.078, η^2^_p_ = 0.072, p = 0.328) (Table 2).

**Table 2:** Results of repeated measures ANOVAs for overall and pooled (epochs) data.

	Variable	df	*F* value	*η*^2^_*p*_	*P* value
Parameters (Overall)	Intensity	2 (28)	1.710	0.109	0.199
	Duration	2 (28)	2.278	0.140	0.121
	Time	3.962^#^ (55.475)	4.614	0.248	0.003*
	Intensity × Duration	4 (56)	0.140	0.010	0.967
	Intensity × Time	28 (392)	1.471	0.095	0.060
	Duration × Time	28 (392)	0.791	0.053	0.770
	Intensity × Duration × Time	56 (784)	1.078	0.072	0.328
Parameters (Pooled)	Intensity	2 (30)	1.925	0.114	0.163
	Duration	2 (30)	2.009	0.118	0.152
	Epoch	3 (45)	14.604	0.493	<0.001*
	Intensity × Duration	4 (60)	0.314	0.020	0.868
	Intensity × Epoch	6 (90)	2.126	0.124	0.058
	Duration × Epoch	6 (90)	1.098	0.068	0.370
	Intensity × Duration × Epoch	5.661^#^ (84.908)	0.832	0.053	0.617
Conditions (Overall)	Condition	9 (126)	2.489	0.151	0.012*
	Time	3.944^#^ (55.210)	4.330	0.236	0.004*
	Condition × Time	126 (1764)	1.155	0.076	0.122
Conditions (Pooled)	Epoch	3 (45)	13.491	0.474	<0.001*
	Condition	4.791^#^ (71.868)	2.686	0.152	0.030*
	Condition × Epoch	27 (405)	1.613	0.097	0.029*

The table shows the results of the repeated measures ANOVAs. First a three-way repeated measures ANOVA was conducted to explore the impact of stimulation duration, and intensity on MEP amplitudes. This analysis was conducted for all obtained time points (overall), and the epoched (pooled) data. A two-way repeated measures ANOVA was conducted with Condition (time-intensity combinations) and Time as within subject factors, for overall, and for pooled data, to explore if the respective real stimulation effects differed from those of the sham intervention. The df column shows degrees of freedom.

*P < 0.05, df = degrees of freedom, η^2^_p_ = partial eta squared. ^#^Greenhouse^−^Geisser correction according to violation of the sphericity condition.

In a second analysis, post-stimulation time was averaged (pooled) into three time bins: early (0–30 mins), late (60–120 mins), and very late (SE-NE) epochs. The ANOVA results show likewise no significant main effect of Intensity (F_(2, 30)_ = 1.925, η^2^_p_ = 0.114, p = 0.163) and Duration (F_(2, 30)_ = 2.009, η^2^_p_ = 0.118, p = 0.152) but a significant main effect of Epoch (F_(3, 45)_ = 14.604, η^2^_p_ = 0.493, p < 0.001). A trend-wise effect was present for the Intensity x Epoch interaction (F_(6, 90)_ = 2.126, η^2^_p_ = 0.124, p = 0.058), but no significant interactions between Intensity and Duration (F_(4, 60)_ = 0.314, η^2^_p_ = 0.020, p = 0.868), Duration and Epoch (F_(6, 90)_ = 1.098, η^2^_p_ = 0.068, p = 0.370), or Intensity, Duration, and Epoch (F_(5.661, 84.908)_ = 0.832, df = 5.661, η^2^_p_ = 0.053, p = 0.617) were revealed.

### After-effects of tDCS on cortical excitability

In these analyses, we investigated the influence of active stimulation conditions on altered cortical excitability relative to sham. Stimulation conditions refer to intensity-duration combinations (Fig. 1).

**Figure 2:**
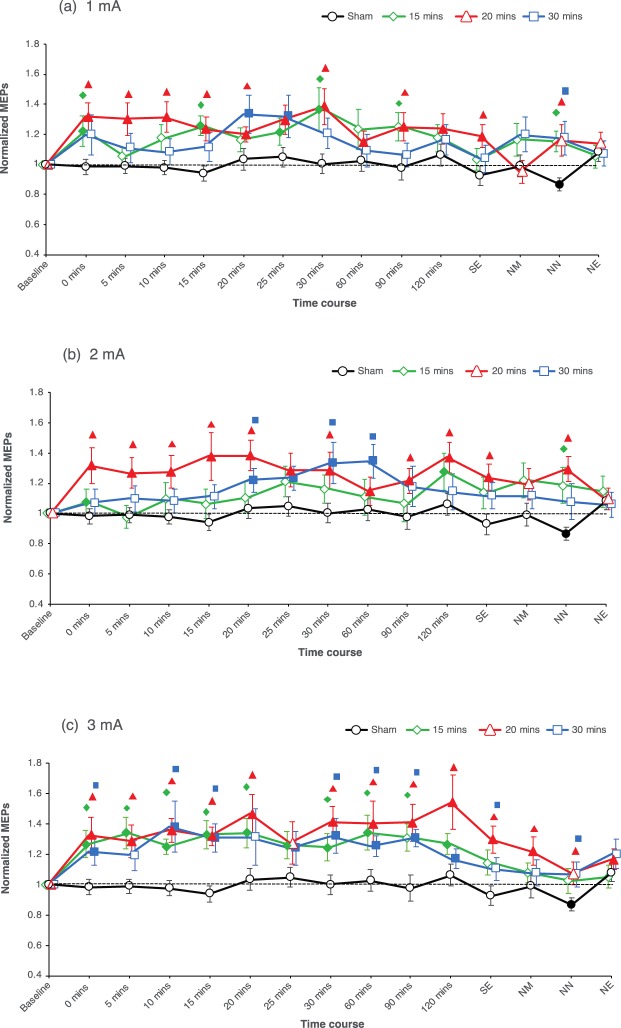
Averaged MEPs post-stimulation for all intervention conditions and monitored time bins. MEPs were obtained before, immediately after tDCS at 5-minute intervals until 30 min after intervention and then every half an hour until 120 mins; same day evening (SE), next morning (NM), next noon (NN) and next evening (NE) for all three tDCS intensity-duration combinations. tDCS resulted in an enhancement of cortical excitability post-stimulation when compared to the respective baselines values, and sham. Sham tDCS did not alter cortical excitability with the exception of significant reduction of MEP amplitudes at NN. The three intensities and durations of stimulation show comparable effects. Comparison of all real tDCS conditions showed no significant differences between the respective induced cortical excitability alterations. Error bars represent standard error of the mean. Filled symbols represent a significant difference of MEP amplitudes compared to the respective baselines. Floating symbols represent significant differences between real and sham stimulation conditions (paired *t* test, two-tailed, *p* < *0.05*).

The two-way repeated measures ANOVA shows significant main effects of Condition (F_(9, 126)_ = 2.489, η^2^_p_ = 0.151, p = 0.012) and Time (F_(3.944, 55.210)_ = 4.330, η^2^_p_ = 0.236, p = 0.004), but no significant interaction between Condition and Time (F_(126, 1764)_ = 1.155, η^2^_p_ = 0.076, p = 0.122) (see Table 2). Post-hoc comparisons of the various time points to baseline show that MEP amplitudes were enhanced or showed trend-wise increases in all, except for the sham condition within the first two-hour period after stimulation (Fig. 2A–C). Compared to their respective baselines, 1 mA–15 min resulted significant cortical excitability enhancements until 30 min (but not for the time points 5, 10, and 20 min), 1 mA–20 min resulted in excitability enhancements that lasted until same day evening (SE), whereas 1 mA–30 min elicited mostly trend-wise excitability enhancements, with significant increases at time points 20 and 25 min after tDCS. Comparisons with sham showed for the 1 mA–15 min stimulation condition significant excitability enhancements at time points 0, 15, 30, 90 min, and next noon (NN) after tDCS. The 1 mA–20 min condition enhanced cortical excitability until NN except at time points 25, 60, 120 min, and next morning (NM) after tDCS, while the 1 mA–30 min condition did not show any significant enhancements in cortical excitability when compared with sham except for the NN time point (Fig. 2A). For the 2 mA stimulation conditions, comparison with baseline showed that 2 mA–15 min did not significantly enhance cortical excitability post-tDCS except for time point 120 min, while 2 mA–20 min resulted excitability enhancements until NN except at the NM timepoint. The 2 mA–30 min condition resulted in delayed enhancements of excitability, with significant increases of MEP amplitudes at timepoints 20 to 60 min post-tDCS. Compared to the sham stimulation condition, 2 mA–15 min did not result in any significant enhancements of cortical excitability post-tDCS except at the NN timepoint, whereas the 2 mA–20 min condition induced an enhancement of MEP amplitudes until NN (except for the 25 min, 60 min, and NM time points). 2 mA–30 min only resulted in MEP enhancements significant at 20, 30, and 60 min post-stimulation when compared to sham (Fig. 2B). For the protocols including 3 mA stimulation intensity, compared to their respective baselines, all conditions induced an enhancement of cortical excitability within the first 2 hours post-tDCS (except at time points 5, and 20 min for 3 mA–30 min, 25 min for 3 mA–20 min, and 90 min for 3 mA–15 min). Likewise, comparisons with sham show that MEP amplitudes were significantly increased in the 3 mA–15 min condition until 90 minutes after stimulation except for time point 25 min. The after-effects of 3 mA–20 min however did last until the NN (except for the 25 min time point), and those of the 3 mA–30 min stimulation protocol lasted also until NN (but not for 5, 20, 25, 120 min, and NM time points), when compared with sham (Fig. 2C). Sham tDCS did not result in any excitability alteration, except a small but significant reduction of MEP amplitudes at the NN timepoint.

Time points post-stimulation were pooled into an early (0–30 min), late (60–120 min), and very late (SE-NE) epochs to control for variability within the time bins.

**Figure 3:**
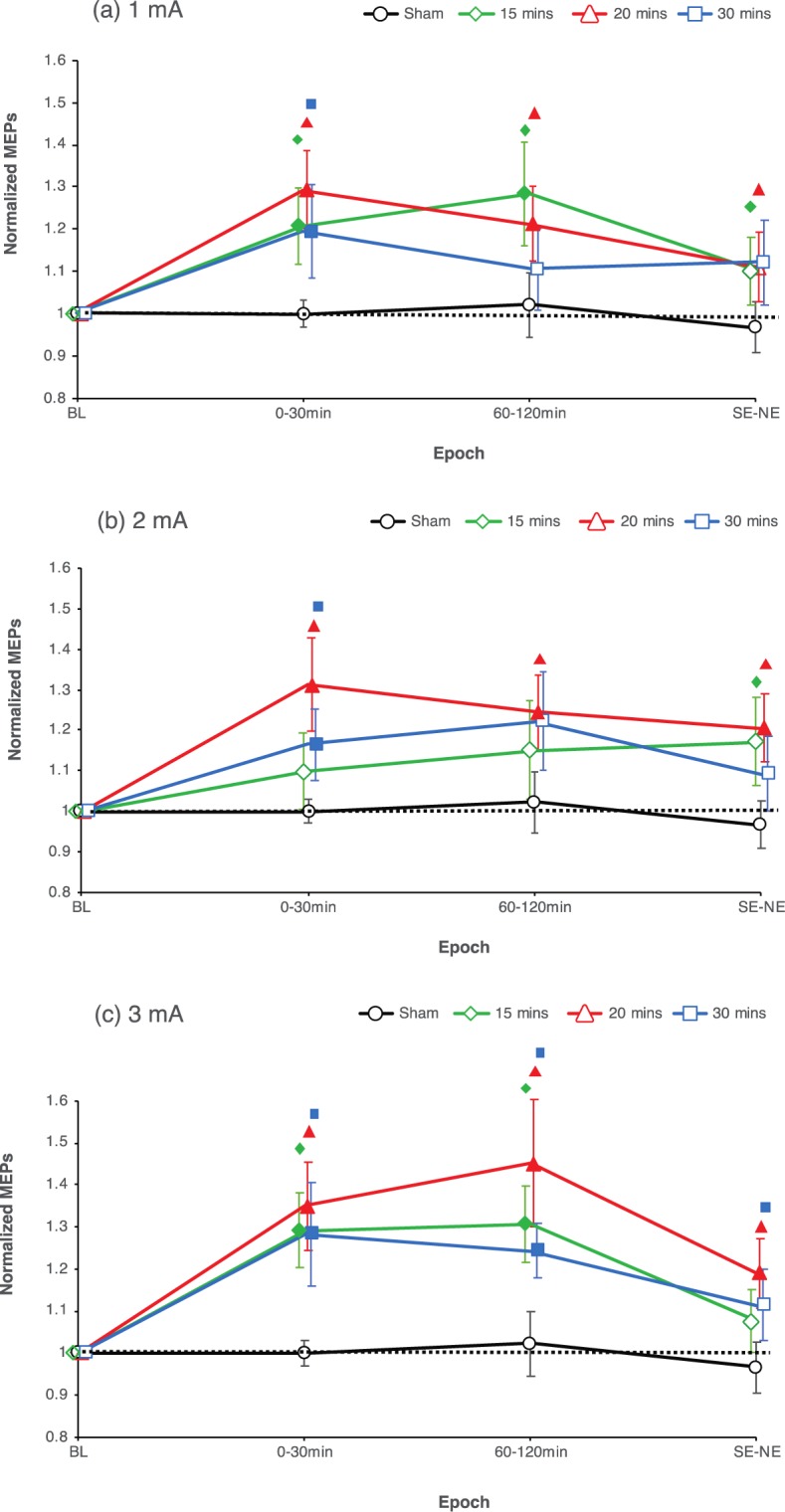
Average MEPs post-stimulation for all intervention conditions and pooled time bins. Post hoc comparisons with sham tDCS indicate that all stimulation conditions resulted in significant cortical excitability enhancements within the first 30 minutes after stimulation (early epoch), except for the 2 mA–15 min condition, which did alter excitability only trend-wise. For the late epoch (60–120 min after stimulation), all conditions except 1 mA–30 mins, 2 mA–15 mins, and 2 mA–30 mins show a cortical excitability increase. Cortical excitability was also enhanced for all conditions in the very late epoch (same day evening to next day evening after stimulation), except for the 1 mA–30 min, 2 mA–30 min, and 3 mA–15 min conditions. Error bars represent standard error of the mean. Filled symbols in the graph represent a significant difference of MEP amplitudes compared to the respective baselines. The floating symbols indicate significant differences between real stimulation and sham conditions (paired t test, two-tailed, p < 0.05).

For the respective pooled data, the ANOVA results indicate significant main effects of Condition (F_(4.791, 71.868)_ = 2.686, η^2^_p_ = 0.152, p = 0.030) and Epoch (F_(3, 45)_ = 13.491, η^2^_p_ = 0.474, p < 0.001), as well as a significant interaction between Condition and Epoch (F_(27, 405)_ = 1.613, η^2^_p_ = 0.097, p = 0.029). Post-hoc comparisons show that all real stimulation conditions resulted in a significant enhancement of cortical excitability when compared to baseline and sham within the first 30 minutes after stimulation (early epoch), except for the 2 mA–15 min stimulation condition (Fig. 3). For the late epochs (60–120 mins after stimulation), all conditions except 1 mA–30 min, 2 mA–15, and 2 mA–30 min showed a significant increase of cortical excitability compared to their respective baselines and sham (Fig. 3). Cortical excitability was significantly enhanced for only the 2 mA–20 min and 3 mA–20 min conditions in the very late epoch (same day evening to next day evening after stimulation) when compared to their respective baselines, but for all conditions when compared to sham except for the 1 mA–30 min, 2 mA–30 min and 3 mA–15 min conditions (Fig. 3).

## Discussion

We explored the parameter space of anodal tDCS in the primary motor cortex of healthy humans. When compared to sham, all stimulation conditions resulted in significant enhancement of MEP amplitudes following anodal tDCS. There were no significant differences between the stimulation intensities, or durations. We however observed a trend-wise increase of excitability enhancements from the lower to the higher current intensities. Participants tolerated the stimulation well, and side-effects did not significantly differ for the respective real stimulation intensities versus sham. Blinding was successful, as participants could not accurately guess the stimulation intensities they received.

With respect to stimulation intensity, this result confirms those of previous studies, which showed only minor or no differences of stimulation intensities between 0.5 and 2 mA^23,26,29,30^. The results of the present study extend the tDCS intensity range showing similar effects for 3 mA. In the current study however, a trend was observed in the intensity domain, with increased effects with higher current intensity. It might thus be that enhancing stimulation intensity further results in higher efficacy of anodal tDCS.

Previous studies with lower current intensities (≤1 mA) and shorter durations (≤13 minutes) have reported an intensity- and duration-dependent effect of anodal tDCS, where increasing the respective parameters resulted in a linear dose-response relationship^8,9^. The relationship between current intensity and induced after-effects of tDCS is however partially non-linear for higher intensities^23^ for cathodal tDCS. Specifically, for anodal tDCS, increasing the current intensity from 1 to 2 mA did not result in a corresponding increase of MEP amplitudes^26,29^. Changes in cortical excitability due to tDCS are calcium-dependent^14,31^, and for LTP induction, calcium concentration within a specific range is required^21^. It could thus be argued that for the previous studies with relatively low tDCS intensities and/or short durations, these were at the lower limit of respective calcium concentrations. Within this range, enhancing calcium concentration should increase efficacy of LTP induction. The calcium concentration range induced by the stronger protocols in the present study, however, might be within the optimal level, and gradual differences at this range might not have a major impact on the level of LTP induction. Furthermore, higher calcium concentration might activate counteracting homeostatic mechanisms, such as the activation of potassium channels, saturation of NMDA receptors, thus limiting the amount of plasticity^22,32,33^. These potential mechanisms require further exploration. In principal accordance, studies using different NIBS techniques have also reported some non-linearities with respect to varying intensity of stimulation^34,35^. Continuous theta burst stimulation (TBS) delivered at an intensity 70% of resting motor threshold (RMT) significantly increased MEP amplitudes, while a decrease of MEP amplitudes was observed when the stimuli were applied at 65% of RMT^35^. In another study, Moliadze and colleagues found a reduction in MEP amplitudes with 0.4 mA transcranial random noise stimulation (tRNS), but an enhancement at 1 mA^34^. The trend-wise increase of cortical excitability observed in the intensity domain (1 vs 2, and 3 mA) could be dedicated to more efficient stimulation of larger and deeper cortical areas with increasing intensity.

In the current study, we did moreover not observe any significant differences between the effects of different stimulation durations. In accordance, previous studies have shown that increasing tDCS duration does not necessarily enhance magnitude or duration of induced plasticity^24,29^. Prolonging the duration of stimulation beyond a critical time point might lead to a saturation of the after-effects, caused probably by calcium overflow^24^. Additionally, we observed that the stimulation duration of 30 minutes, for both, 1 and 2 mA intensities, resulted in delayed enhancements of cortical excitability post-tDCS (Figs. 2 and 3A,B). Previous studies with similar stimulation protocols already described delayed enhancements of cortical excitability post-tDCS^23,36^. This could reflect longer calcium dynamics at the postsynaptic membrane, causing activation of potassium channels which might have led to transient counterbalancing plasticity induction^22,32^. The lack of a significant excitability enhancement at some time points in some conditions when compared to their respective baselines and/or the sham stimulation condition, in spite of a uniform trend also in these conditions is probably due to response variability, which has been reported in previous tDCS studies^27,37^, and is present also in the individual data of the present study (Suppl. Material S1-Fig. 1A–J). This variability is much less obvious in the pooled data, where each epoch relies on a larger number of MEPs (Fig. 3A–C). The significant effects seen at the time point NN for almost all protocols when compared to sham could reflect, at least in part, the significant decrease in MEP observed for sham at this time point, as no other real stimulation condition except 2 mA–20 min showed any enhancement in MEP at this time point when compared to their respective baselines, and should thus be taken with caution.

Importantly, except for the 3 mA–20 min and 2 mA–20 min conditions, none of the stimulation protocols we probed induced relevant late-phase plasticity, which we define as respective enhancements of cortical excitability compared to baseline and sham and lasting for more than a few hours after intervention (Fig. 3A–C). We chose this relatively strict criterion because for one of the late MEP measures (NN) we obtained a significant reduction of MEP amplitudes, which was most probably artefactual (Fig. 2A–C). To compensate for this, we interpreted late-phase MEP amplitude alterations only as relevant if these differed also significantly vs the respective baseline MEPs. It should however be added that though both stimulation conditions induced late-phase plasticity according to this criterion, the amplitudes were moderate. The lack of all other protocols in this study to induce relevant late-phase plasticity is in accordance with other studies in the field^14,20^, that have demonstrated in animal as well as human models that single session stimulation protocols are not well suited for inducing late-phase plasticity. In animal studies, single plasticity-inducing interventions resulted only in early-phase LTP^38,39^, whereas repeated intervention within an interval of 30 minutes resulted in late-phase LTP, lasting for several hours longer^40,41^. tDCS with protocols comparable to those efficient in animal models of late phase plasticity induction, including repetition of stimulation within a critical time window of 30 min, or additional pharmacological interventions, result in late phase effects also in humans. Repetition intervals of 3 or 20 minutes enhanced cortical excitability for at least 24 hours after stimulation^24^. Likewise, the serotonin and noradrenaline reuptake inhibitors citalopram and reboxetine enhance the after-effects of anodal tDCS for up to 24 hours^42,43^. Thus, taking the relatively short-lived after-effects of the extended stimulation protocols used in the present study into account, combined interventions might be required, if late-phase plasticity is aimed for. It would nevertheless be interesting to explore if the newly introduced intensified protocols result in more efficient late-phase plasticity, if these are used in respective repeated stimulation designs.

It should be noted that, although we did not find any significant differences between the three intensities and durations tested in the present experiment in healthy participants for the primary motor cortex, this does not exclude that higher intensities, or longer duration of stimulation improve efficacy of stimulation in specific settings, which might include specific brain states, or architecture. 2 and 3 mA tDCS enhanced treatment outcomes compared to 1 mA in patients with schizophrenia^44,45^, and 2 mA tDCS had a better effect than 1 mA for improving working memory task performance in Parkinson’s disease^46^. In neuropsychiatric disorders, specific changes of cortical structure and function such as activity alterations of neurotransmitters, cerebral atrophy, and other alterations could shift the parameter range for efficient stimulation, and thus the respective parameter range must be explored systematically in the respective conditions.

This study should be interpreted within the context of a few limitations. Our sample size of 16 young healthy participants is relatively small and homogeneous, thus results obtained from this group cannot be perfectly extrapolated to patient and elderly populations. The results of this study were obtained at the group level, and as shown in the Supplemental Material, interindividual variability was present, similar to previous studies^8,9,37^. Consequently, results might differ between individuals. We did not investigate mechanistic details of tDCS-induced plasticity in this study, or other factors that might contribute to tDCS-induced neuroplasticity, such as genetic factors. Finally, the transferability of these protocols from the motor to other cortical areas has to be explored in future studies.

In conclusion, we extended the parameter space of anodal tDCS with regard to intensity and duration for up to 3 mA and 30 mins respectively and found tDCS effects in this protocol range (1–3 mA; 15–30 mins) to be relatively similar, and robust to gradual protocol changes. The highest intensity applied in this study (3 mA) showed trend-wise superior effects. The similarity of anodal tDCS effects at the group level encourages the use of protocols within this parameter range. This expansion, and systematic evaluation of the stimulation parameter range thus help to define optimal stimulation protocols for experimental and clinical use.

## Methods

### Participants

Sixteen non-smoking right-handed healthy volunteers (9 females, mean age 26.5 ± 2.2 (SD) years) participated in the study. Participants were medically examined to ascertain their overall health state. None of the volunteers participating in the experiment had any history of any neurological or psychiatric disorders, or brain implants. During the course of the experiment, participants were instructed not to drink coffee at least for two hours before each session. We used a single-blind study design. The participants were blinded for all conditions of the experiment, and the order of all conditions was randomized. This study was approved by the ethics committee of Leibniz Research Centre for Working Environment and Human Factors (IfADo) and agrees with the provisions of the Helsinki Declaration^47^. Each participant gave written informed consent after the general objectives of the experiment were explained to them. All participants were naïve to TMS and tDCS prior to participation in this study. Beyond screening of exclusion criteria, no other selection criteria were applied.

### tDCS

tDCS was applied with a battery-powered stimulator (neuroCare, Ilmenau, Germany) delivering constant direct current (maximum DC output of 4 mA) through a pair of carbonated rubber electrodes covered by saline-soaked sponges, each measuring 35 cm^2^ (7 × 5 cm). The anode was placed over the left primary motor cortex (M1), over the hotspot of the abductor digiti minimi muscle (ADM) representation, as determined by TMS, while the return electrode was positioned over the right supra-orbital area. Depending on the experimental condition, tDCS was applied for 15, 20, or 30 minutes with intensities of 1, 2, or 3 mA. For each stimulation intensity, current was ramped up for 10 seconds at the beginning and ramped down for 10 seconds at the end of stimulation. For sham stimulation, 1 mA tDCS was applied for 15 seconds (current was ramped up for 10 seconds, 15 seconds of stimulation, after which current was ramped down for 10 seconds), but the tDCS electrodes were kept on the participants’ head for 15 minutes to mimic a true experimental condition.

### Cortical excitability measurements

A TMS stimulator (Mag and More, Munich, Germany) delivered magnetic pulses at 0.20–0.25 Hz through a figure-of-8 coil (diameter of 70 mm, maximum magnetic field of 2 Tesla). The coil was held tangentially to the skull with the handle pointing backwards at 45 degrees to the midline. The ‘motor hotspot’, the optimal area of the primary motor cortex representing the ADM was determined as the region where TMS of a given intensity elicited the highest average MEPs. Electromyography (EMG) electrodes attached to the ADM in a belly tendon montage recorded MEPs induced by single pulse biphasic TMS. Analogue EMG signals were then sampled at 5 kHz (CED, Cambridge, UK), amplified and band pass-filtered at 2 Hz–2 kHz (Digimeter, Hertfordshire, UK) using the Signal software (version 4.0), and stored offline for further analyses.

### Experimental procedure

Participants were comfortably seated in a reclining chair, with an inflatable pillow wrapped around their necks to stabilize the head position. Each experimental session began in the morning at about 09:30. First, the motor hotspot was identified as described above and marked. TMS stimulus intensity was then adjusted to produce an average peak-to-peak 1 mV MEP amplitude, defined as baseline TMS Intensity, and kept constant throughout the remaining experimental session. 25 MEPs were obtained as baseline cortical excitability measure. tDCS was then applied according to the respective experimental condition. 25 MEPs were recorded with the same baseline TMS intensity immediately after tDCS, and every 5 minutes for up to 30 minutes, and then every 30 minutes until 2 hours after intervention. Additionally, cortical excitability was measured at the same day evening (SE) (approximately 7 hours after tDCS), next morning (NM) (23–24 hours after tDCS), next noon (NN) (about 28 hours after tDCS), and next evening (NE) (about 32 hours after tDCS). To minimize discomfort associated with higher stimulation intensities, a topical analgesic cream, EMLA (2.5% lidocaine and 2.5% prilocaine) was applied to the scalp before each stimulation session.

After each session, a questionnaire^48,49^ was administered to gain information on how participants rated side-effects of tDCS, and to guess the stimulation intensity they received (sham, 1, 2, or 3 mA).

### Calculations and statistical analyses

The peak-to-peak amplitudes of MEPs at each time point post-stimulation were measured, averaged, and normalized to the baseline for each individual. To exclude differences between the baseline MEP and baseline TMS intensity between sessions, a one-way repeated measures ANOVA was conducted with baseline MEP and baseline TMS as dependent variables and session as a within-subject factor.

To investigate the effect of the tDCS parameters – stimulation intensity, and duration - on cortical excitability, a three-way repeated measures ANOVA was conducted with ‘Intensity’, ‘Duration’, and ‘Time’ as within subject factors and mean normalized MEPs as dependent variable. Time refers to the overall stimulation time frame, from baseline up until the next day evening (15 time points). For further analysis, and to compensate for variability between single time bins, we conducted another ANOVA for pooled time bins. Here, post-stimulation time bins were pooled into three time frames – early (0–30 min), late (60–120 min) and very late (SE-NE). Averages were computed over all MEPs of each binned time point: early epoch (0–30 min, 6 time points – including 150 MEP), late epoch (60–120 min, 3 time points – 75 MEP), and very late epoch (SE-NE, 4 time points – 100 MEP). A three-way ANOVA was conducted for the pooled (epoched) data with ‘Intensity’, ‘Duration’ and ‘Epoch’ as within subject factors and mean normalized MEPs as dependent variable.

To investigate if the real stimulation conditions altered excitability relative to sham, a two-way repeated measures ANOVA was conducted with ‘Condition’ (10 levels) and ‘Time’ (15 levels) as within-subject factors and normalized mean MEP amplitude values at each time point as dependent variable. Additionally, a two-way repeated measure ANOVA was conducted for pooled data, with ‘Condition’ and ‘Epoch’ as within-subject factors and mean normalized MEPs as dependent variable, as described above.

For all ANOVAs, Mauchly’s test of sphericity was conducted, and where necessary, the Greenhouse-Geisser correction was applied. In case of significant results of the ANOVAs, exploratory post-hoc Student’s t-tests (paired samples, two-tailed, *p* < *0.05*, not corrected for multiple comparisons) were conducted to determine significant differences between baseline and post-tDCS MEPs, between conditions of active stimulation and sham, and between respective active conditions of stimulation. Exploratory post-hoc tests were not corrected for multiple comparisons to avoid a relevant enhancement of type II errors, according to Perneger^50^, and Feise^51^. This is also in accordance with other studies in the field, and thus increases inter-study comparability^52,53^.

All analyses were carried out using SPSS, version 24 (IBM SPSS Statistics, New York, USA).

Calculations and statistics for the side-effect, and blinding questionnaires are described in the Supplemental Material.

## Supplementary information

Supplementary Material
