# Supplementary material for: Expanding the parameter space of anodal transcranial direct current stimulation of the primary motor cortex
Source: Sci Rep. 2019 Dec 3;9:18185. doi: 10.1038/s41598-019-54621-0 (PMC6890804; doi:10.1038/s41598-019-54621-0)
Supplement: Supplementary file 1 — Supplementary Material [file 41598_2019_54621_MOESM1_ESM.docx]

**Title: Expanding the parameter space of anodal transcranial direct current stimulation of the primary motor cortex.**

**Authors: Desmond Agboada^1,2^; Mohsen Mosayebi Samani^1,3^; Asif Jamil^1^; Min-Fang Kuo^1^; Michael A. Nitsche*^1,4^.**

**S1. Individual time series data.**

**Figure 1**. Individual data of all 16 participants (labelled as P01, P02,…, P16) for all ten stimulation conditions (A-J)

A

B

C

D

E

F

G

H

I

J

**S2. Side-effects of tDCS**

| Time | Side-effects | Sham | 1mA-15min | 1mA-20min | 1mA-30min | 2mA-15min | 2mA-20min | 2mA-30min | 3mA-15min | 3mA-20min | 3mA-30min |
| --- | --- | --- | --- | --- | --- | --- | --- | --- | --- | --- | --- |
| During tDCS | Visual | 0.19  ±0.75 | 0.13  ±0.34 | 0.44  ±1.09 | 0.19  ±0.40 | 0.06  ±0.25 | 0.50  ±0.82 | 0.50  ±0.89 | 0.31  ±0.60 | 0.50  ±0.89 | 0.13  ±0.34 |
|  | Itching | 1.06  ±1.57 | 0.75  ±1.13 | 0.69  ±1.01 | 0.94  ±1.24 | 1.44  ±1.50 | 1.13  ±1.31 | 1.38  ±1.45 | 1.13  ±1.59 | 1.69  ±1.82 | 0.81  ±0.98 |
|  | Tingling | 1.00  ±1.31 | 0.93  ±1.24 | 1.00  ±1.15 | 1.25  ±0.93 | 1.44  ±1.20 | 1.06  ±0.93 | 1.31  ±1.25 | 0.94  ±1.00 | 1.25  ±1.06 | 1.06  ±0.85 |
|  | Burning | 1.19  ±1.26 | 1.00  ±1.41 | 1.06  ±1.43 | 1.31  ±1.25 | 1.69  ±1.35 | 1.31  ±1.58 | 1.88  ±1.63 | 2.06  ±1.77 | 1.69  ±1.85 | 1.69  ±1.45 |
|  | Pain | 0.63  ±1.26 | 0.44  ±0.69 | 0.44  ±0.81 | 0.25  ±0.58 | 0.38  ±0.81 | 0.75  ±1.06 | 1.00  ±1.37 | 1.00  ±1.15 | 0.75  ±1.24 | 0.88  ±1.26 |
| 24 hours after tDCS | Redness | 0.44  ±1.26 | 0.19  ±0.54 | 0.19  ±0.54 | 0.31  ±0.87 | 0.13  ±0.34 | 0.31  ±0.79 | 0.38  ±1.02 | 0.25  ±1.00 | 0.31  ±0.79 | 0.19  ±0.54 |
|  | Headache | 0.56  ±1.26 | 0.50  ±0.82 | 0.81  ±1.04 | 0.56  ±0.81 | 0.63  ±1.31 | 0.69  ±1.30 | 0.94  ±1.34 | 0.50  ±0.89 | 0.69  ±0.95 | 0.56  ±0.81 |
|  | Fatigue | 1.06  ±1.44 | 0.94  ±1.24 | 1.25  ±1.44 | 1.25  ±1.13 | 0.81  ±1.11 | 1.00  ±1.15 | 1.19  ±1.28 | 0.81  ±1.04 | 1.06  ±1.06 | 0.94  ±1.06 |
|  | Concentration | 0.93  ±1.29 | 0.69  ±1.08 | 0.69  ±1.01 | 0.56  ±0.73 | 0.50  ±0.63 | 1.13  ±1.59 | 1.19  ±1.28 | 0.81  ±1.42 | 1.06  ±1.12 | 0.81  ±1.11 |
|  | Nervousness | 0.38  ±1.29 | 0.19  ±0.40 | 0.63  ±1.15 | 0.25  ±0.77 | 0.38  ±0.72 | 0.50  ±1.03 | 0.31  ±1.01 | 0.38  ±0.89 | 0.31  ±1.01 | 0.69  ±1.20 |
|  | Sleep | 0.31  ±0.79 | 0.75  ±1.34 | 0.13  ±0.34 | 0.25  ±0.68 | 0.25  ±0.58 | 0.44  ±0.81 | 0.50  ±0.97 | 0.31  ±0.87 | 0.25  ±0.58 | 0.25  ±0.44 |

**Table 1**. Side-effects commonly reported by participants in tDCS experiments.

This table shows the average values and standard deviations of perceived intensity of known side-effects of tDCS as measured on a Likert scale (0-5, with 0 referring to absence of side-effect, and 5 as highest severity of side-effect). Mean values ± standard deviation.

| Time | Side-effects | df | F value | η^2^_p_ | p |
| --- | --- | --- | --- | --- | --- |
| During tDCS | Visual | 9 | 2.269 | 0.131 | 0.021* |
|  | Itching | 4.101 | 1.742 | 0.104 | 0.151 |
|  | Tingling | 9 | 0.826 | 0.052 | 0.538 |
|  | Burning | 5.061 | 2.347 | 0.135 | 0.048* |
|  | Pain | 3.882 | 1.613 | 0.097 | 0.185 |
| 24 hours after tDCS | Redness | 9 | 1.107 | 0.069 | 0.362 |
|  | Headache | 3.635 | 0.465 | 0.030 | 0.744 |
|  | Fatigue | 4.640 | 0.641 | 0.041 | 0.658 |
|  | Concentration | 3.675 | 1.498 | 0.091 | 0.219 |
|  | Nervousness | 9 | 0.808 | 0.051 | 0.609 |
|  | Sleep | 2.887 | 1.069 | 0.067 | 0.370 |

**p < 0.05*

**Table 2.** One-factorial ANOVAs were conducted for all tDCS side-effects, with stimulation condition as within subject factor, and the values of the Likert scale as dependent variable.

Visual and burning sensations showed significant main effects during tDCS. No side-effect reported 24 hours after tDCS differed significantly between conditions. Post-hoc comparisons of sham versus active stimulation conditions showed no significant differences of perception of visual or burning sensations.

To investigate the influence of visual and burning sensations on the after-effects of tDCS, a Pearson correlation coefficient was calculated between the reported side-effects, and pooled MEP post-tDCS. There was no significant correlation between visual sensations and tDCS effects in the early (Pearson r = 0.53, p = 0.11), late (Pearson r = 0.48, p = 0.16) and very late (Pearson r = 0.30, p = 0.40) epochs of all ten conditions. Also burning sensations did not show any significant correlation with tDCS after-effects (Early epoch, Pearson r = 0.17, p = 0.62; Late epoch, Pearson r = 0.35, p = 0.31; and very late, Pearson r = 0.13, p = 0.70).

**S3. Blinding of Participants to stimulation intensity**

|  | Intensity | guessed | by | participants |  |  |
| --- | --- | --- | --- | --- | --- | --- |
|  |  | Sham | 1 mA | 2 mA | 3 mA | Total |
|  | Sham | 5 | 10 | 0 | 1 | 16 |
| Actual | 1 mA | 11 | 23 | 13 | 1 | 48 |
| Intensity | 2 mA | 9 | 23 | 14 | 2 | 48 |
|  | 3 mA | 4 | 21 | 15 | 8 | 48 |

**Table 3**. Table showing the frequency of actual tDCS versus guessed intensities. The rows represent the actual intensity applied in the respective experiment, whereas the columns represent the perceived intensity. The difference between the frequency of sham and active stimulation conditions (1, 2, and 3 mA) is due to the presence of only one sham condition as opposed to 3 stimulation intensities.

| Chi-Square | 22.500 |
| --- | --- |
| df | 1 |
| p | <0.001* |

* *P < 0.05*

**Table 4**: results of the Chi-square test of responses (guessed vs actual) for all conditions of stimulation. There was a significant heterogeneity between responses of participants in the perception of tDCS intensities received; χ^2^ (1, N = 160) = (22.50), p < 0.001.

|  | Sham | 1 mA | 2 mA | 3 mA |
| --- | --- | --- | --- | --- |
| Chi-square | 2.250 | 0.083 | 8.333 | 21.333 |
| df | 1 | 1 | 1 | 1 |
| p | 0.210 | 0.885 | 0.006* | < 0.001* |

* *P < 0.05*

**Table 5**. Chi-Square table showing the blinding data of participants for each intensity domain including sham.

Participants were not able to accurately guess the stimulation they received in the sham and 1 mA conditions, χ^2^ (1, N = 16) = (2.250), p = 0.210, χ^2^ (1, N = 48) = (0.083), p = 0.885. There were however statistically significant differences between perceived and actual intensities with respect to 2, χ2 (1, N = 48) = (8.333), p = 0.006, and 3 mA actual stimulation intensities χ2 (1, N = 48) = (21.333), p < 0.001.

|  | Sham | 1mA-15min | 1mA-20min | 1mA-30min | 2mA-15min | 2mA-20min | 2mA-30min | 3mA-15min | 3mA-20min | 3mA-30min |
| --- | --- | --- | --- | --- | --- | --- | --- | --- | --- | --- |
| Chi-Square | 2.250 | 1.000 | 0.000 | 0.250 | 1.000 | 9.000 | 1.000 | 6.250 | 12.250 | 4.000 |
| df | 1 | 1 | 1 | 1 | 1 | 1 | 1 | 1 | 1 | 1 |
| p | 0.210 | 0.454 | 1.000 | 0.804 | 0.454 | 0.004* | 0.454 | 0.021* | 0.001* | 0.077 |

* *P < 0.05*

**Table 6**. Chi-Square table showing the perception of current intensity received (actual vs guessed) for each of the 10 sessions. There was a significant difference between guessed and the actual intensity received in the 2mA-20min, 3mA-15min, and 3mA-20min conditions.

| Condition | Sham | 1mA-15min | 1mA-20min | 1mA-30min | 2mA-15min | 2mA-20min | 2mA-30min | 3mA-15min | 3mA-20min | 3mA-30min | Total |
| --- | --- | --- | --- | --- | --- | --- | --- | --- | --- | --- | --- |
| Wrongly guessed | 11 | 10 | 8 | 7 | 10 | 14 | 10 | 13 | 15 | 12 | 110 |
| Correctly guessed | 5 | 6 | 8 | 9 | 6 | 2 | 6 | 3 | 1 | 4 | 50 |
| Total | 16 | 16 | 16 | 16 | 16 | 16 | 16 | 16 | 16 | 16 | 160 |

**Table 7**. Frequency table of perception of the stimulation intensity received. As can be seen from the results, for the 2, and 3 mA actual stimulation intensities, the probability to guess the correct stimulation intensity was less than chance level. Thus it can be concluded that blinding was not compromised also with these stimulation intensities.

**S4. ANOVA of Baseline and TMS Intensity**

| Factor | df | F | η^2^_p_ | p |
| --- | --- | --- | --- | --- |
| Baseline MEP | 9 (135) | 1.146 | 0.071 | 0.335 |
| Baseline TMS Intensity | 3.469 (52.028)**^#^** | 0.634 | 0.041 | 0.619 |

***^#^****Greenhouse-Geisser correction according to violation of the sphericity condition.*

**Table 8**. Baseline MEPs and TMS intensity.

The ANOVA results show no significant main effects for baseline MEP size (F_(9, 135)_ = 1.146, df = 9, η^2^p = 0.071, p = 0.335), and baseline TMS intensity (F_(3.469, 52.028)_ = 0.634, η^2^p = 0.041, p = 0.619) between the 10 sessions of the experiment.
